# Complete chloroplast genome sequence of the wild diploid potato relative, *Solanum brevicaule*

**DOI:** 10.1080/23802359.2019.1693292

**Published:** 2019-11-20

**Authors:** Tae-Ho Park

**Affiliations:** Department of Horticulture, Daegu University, Gyeongsan, South Korea

**Keywords:** Chloroplast, genome, genome sequence, *Solanum brevicaule*

## Abstract

*Solanum brevicaule* is a wild tuber-bearing species belonging to Solanaceae family. The complete chloroplast genome of *S. brevicaule* was constituted by *de novo* assembly using a small amount of whole genome sequencing data. The chloroplast genome of *S. brevicaule* was the circular DNA molecule with a length of 155,531 bp and consisted of 85,981 bp of large single copy, 18,352 bp of small single copy, and 25,599 bp of a pair of inverted repeat regions. A total of 158 genes were annotated including 105 protein-coding genes, 45 tRNA genes and eight rRNA genes. Maximum likelihood phylogenetic analysis with 30 *Solanaceae* species revealed that *S. brevicaule* is grouped with other *Solanum* species including *S. tuberosum*.

One of wild *Solanum* species, *Solanum brevicaule*, belongs to a taxonomically confusing group (called *S. brevicaule* Bitter complex) within *Solanum* sect. *Petota*. The species is originating from the south of Peru to the north of Argentina and its ploidy level is diverse from diploid to hexaploid (Miller and Spooner 1999; Hardigan et al. 2015). The plant material used in this study is wild tuber-bearing diploid originating from Argentina. Its EBN (Endosperm Balanced Number) value of two theoretically makes it directly crossable for breeding purposes with cultivated tetraploid potatoes (*S. tuberosum*) (Hawkes 1990; Ortiz and Ehlenfeldt 1992; Cho et al. 1997; Spooner et al. 2014). The species was identified as a source of resistance to a nematode, *Globodera pallida* and a soft rot disease caused by *Pectobacterium carotovorum* (Jackson et al. 1988; Chung et al. 2011). As *S. brevicaule* is taxonomically confusing and can be used for potato breeding, therefore, the information of plastid genome of the wild species obtained in this study will provide an opportunity to investigate more detailed evolutionary and breeding aspects.

The *S. brevicaule* (PI205394) was sampled from Highland Agriculture Research Institute, South Korea (37.7° N, 128.7° E). An Illumina paired-end (PE) genomic library was constructed with total genomic DNA according to the PE standard protocol (Illumina, San Diego, USA) and sequenced using an Illumina HiSeq2000 at Macrogen (http://www.macrogen.com/kor/). Low-quality bases with raw scores of 20 or less were removed and approximately 2.5 Gbp of high-quality of PE reads were assembled by a CLC genome assembler (CLC Inc, Rarhus, Denmark) (Kim et al. 2015). The reference chloroplast genome sequence of *S. berthaultii* (KY419708, Park 2017; Kim et al. 2018) was used to retrieve principal contigs representing the chloroplast genome from the total contigs using Nucmer (Kurtz et al. 2004). The representative chloroplast contigs were arranged in order based on BLASTZ analysis (Schwartz et al. 2003) with the reference sequence and connected to a single draft sequence by joining overlapping terminal sequences. DOGMA (Wyman et al. 2004) and BLAST searches were used to predict chloroplast genes.

The complete chloroplast genome of *S. brevicaule* (GenBank accession no. MK036507) was 155,531 bp in length including 25,599 bp inverted repeats (IRa and IRb) regions separated by small single copy (SSC) region of 18,352 bp and large single copy (LSC) region of 85,981 bp with the typical quadripartite structure of most plastids, and the structure and gene features were typically identical to those of higher plants. A total of 158 genes with an average size of 583.0 bp were annotated including 105 protein-coding genes with an average size of 764.6 bp, 45 tRNA genes and 8 rRNA genes with an average size of 223.4 bp. An overall GC content was 37.25%.

Phylogenetic analysis was performed using chloroplast coding sequences of *S. brevicaule* and 30 published species in Solanaceae family by a maximum likelihood method in MEGA 6.0 (Tamura et al. 2013). According to the phylogenetic tree, *S. brevicaule* belonged to the same clade in *Solanum* species as expected (Figure 1).

**Figure 1.**
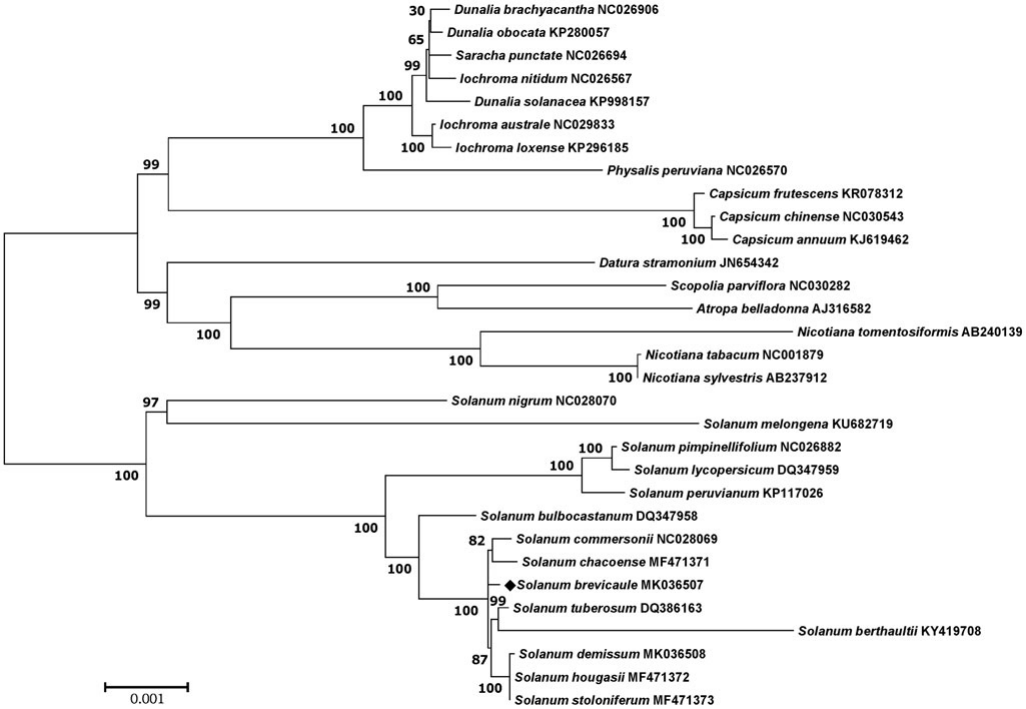
Maximum likelihood phylogenetic tree of *S. brevicaule* with 30 species belonging to the Solanaceae based on chloroplast protein coding sequences. Numbers in the nodes are the bootstrap values from 1000 replicates.
